# Supplementary material for: Impact of Multi-Professional Intervention on Health-Related Physical Fitness and Biomarkers in Overweight COVID-19 Survivors for 8 and 16 Weeks: A Non-Randomized Clinical Trial
Source: Healthcare (Basel). 2024 Oct 14;12(20):2034. doi: 10.3390/healthcare12202034 (PMC11506869; doi:10.3390/healthcare12202034)
Supplement: Supplementary file 1 [file healthcare-12-02034-s001.zip › healthcare-3244907-supplementary.pdf]

Supplementary data are presented separately by sex (male *vs.* female) without considering symptoms and respective COVID-19 symptoms. Supplementary Table 1 shows Anthropometry and body composition responses pre-test, after 8, and after 16 weeks of intervention in the three COVID-19 survivors' groups per sex (males and females).

Supplementary Table S1. Anthropometry and body composition responses pre-test, after 8, and after 16 weeks of intervention in the three COVID-19 survivors' groups per sex (males and females).

Variables	Males (*n* = 32)			Females (*n* = 27)		
	**Pre-test**	**Post-8W**	**Post-16W**	**Pre-test**	**Post-8W**	**Post-16W**
Body mass (kg)	97.3 ± 21.4	96.7 ± 21.0	94.7 ± 24.6	76.1 ± 13.1	74.2 ± 14.1	76.5 ± 12.8
BMI (kg/m^2)	31.3 ± 5.5	31.2 ± 5.3	31.0 ± 5.2	30.2 ± 5.2	30.3 ± 5.1	30.3 ± 5.3
AC (cm)	108.4 ± 15.1	106.8 ± 14.1	107.1 ± 14.9	98.5 ± 11.8	97.6 ± 10.8	97.5 ± 10.7
FMM (kg)	61.4 ± 9.8	60.4 ± 11.1	62.4 ± 11.1	42.4 ± 5.2	42.7 ± 5.0	42.9 ± 5.1
SMM (kg)	34.3 ± 5.9	34.3 ± 6.1	35.0 ± 6.6	23.1 ± 3.1	23.3 ± 2.9	23.4 ± 3.0
FM (kg)	35.9 ± 14.2	35.4 ± 13.6	35.4 ± 13.0	33.7 ± 10.2	33.7 ± 10.1	33.6 ± 9.8
BFP (%)	36.2 ± 7.4	35.5 ± 7.6	34.5 ± 7.6	43.5 ± 7.0	43.2 ± 6.9	43.2 ± 6.5

Note: Data was described by the mean and standard deviation (±). W = weeks; BMI = body mass index; AC = abdominal circumference; FFM = fat-free mass; SMM = musculoskeletal mass; FM = fat mass; BFP = body fat percentage.

Supplementary Table S2 presents the physical and cardiorespiratory fitness responses pre-test, after 8 and 16 weeks of intervention in the three COVID-19 survivors' groups per sex (males and females).

Supplementary Table S2. Physical and cardiorespiratory fitness responses pre-test, after 8, and after 16 weeks of intervention in the three COVID-19 survivors' groups per sex (males and females).

Variables	Males (*n* = 32)			Females (*n* = 27)		
	**Pre-test**	**Post-8W**	**Pre-test**	**Post-8W**	**Pre-test**	**Post-8W**
MIHS-R (kgf)	38.7 ± 9.8	41.8 ± 10.2	41.6 ± 11.0	21.4 ± 5.4	24.4 ± 4.6	26.0 ± 4.8
MIHS-L (kgf)	36.0 ± 9.4	39.4 ± 10.5	40.8 ± 11.3	20.7 ± 5.3	23.4 ± 5.5	24.8 ± 4.8
Sit and reach (cm)	20.4 ± 9.2	22.6 ± 10.1	24.4 ± 8.8	24.7 ± 7.4	26.7 ± 7.0	29.8 ± 5.3
MILTS (kg)	114.5 ± 35.9	123.5 ± 34.3	122.2 ± 36.0	60.2 ± 16.6	72.0 ± 15.9	76.9 ± 20.1
Push-up (reps/min)	16.0 ± 9.5	19.7 ± 11.3	24.9 ± 13.9	19.3 ± 7.1	23.8 ± 7.5	25.0 ± 11.0

Abdominal strength-endurance (reps/min)	18.9 ± 8.3	23.0 ± 9.2	26.8 ± 12.6	13.0 ± 7.0	17.0 ± 8.3	20.0 ± 9.6
Sit and stand (reps/min)	16.5 ± 5.0	19.6 ± 7.2	20.5 ± 6.6	15.5 ± 4.5	18.1 ± 3.3	20.1 ± 3.8
6MWT						
VO_2peak (mL/kg/min)	17.7 ± 4.3	18.7 ± 4.5	19.2 ± 4.3	15.6 ± 3.5	16.6 ± 3.6	17.2 ± 3.6
Distance (m)	554.9 ± 85.4	609.1 ± 97.7	650.7 ± 98.5	489.7 ± 80.7	533.6 ± 76.6	546.9 ± 73.1
SpO_2 final (%)	95.0 ± 2.6	95.6 ± 2.1	95.2 ± 2.5	96.3 ± 2.0	96.8 ± 1.9	95.8 ± 1.9
Final heart rate (bpm)	130.2 ± 26.2	131.7 ± 27.7	134.3 ± 24.3	117.0 ± 21.5	128.2 ± 22.9	124.2 ± 16.6
SBP pre-test (mmHg)	127.4 ± 12.6	129.6 ± 12.4	128.0 ± 15.6	123.7 ± 11.8	127.5 ± 16.8	124.4 ± 11.0
SBP final (mmHg)	144.2 ± 19.3	148.7 ± 11.7	149.4 ± 14.4	141.5 ± 18.5	147.2 ± 14.0	135.3 ± 16.1
DBP pre-test (mmHg)	81.3 ± 11.2	84.2 ± 11.0	76.1 ± 10.6	76.7 ± 9.6	81.5 ± 10.8	71.1 ± 8.3
DBP final (mmHg)	85.2 ± 13.1	87.3 ± 11.4	80.3 ± 11.4	85.2 ± 11.6	88.9 ± 12.8	72.7 ± 10.2

Note: Data was described by mean and standard deviation (±). W = weeks; SBP = systolic blood pressure; DBP = diastolic blood pressure; MIHS = maximal isometric handgrip strength; R = right side; L = left side; MILTS = maximal isometric lumbar-traction strength; 6MWT = 6-minute walk test; VO_2peak = peak oxygen consumption.

Supplementary Table S3 presents biochemical parameters responses pre-test, after 8, and after 16 weeks of intervention in the three COVID-19 survivors' groups per sex (males and females).

Supplementary Table S3. Biochemical parameters responses pre-test, after 8, and after 16 weeks of intervention in the three COVID-19 survivors' groups per sex (males and females).

Variables	**Males (n = 32)**			**Females (n = 27)**		
	**Pre-test**	**Post-8W**	**Post-16W**	**Pre-test**	**Post-8W**	**Post-16W**
TC (mg/dL)	182.1 ± 42.2	156.9 ± 34.7	161.3 ± 38.9	193.4 ± 63.4	164.1 ± 38.7	166.3 ± 33.0
LDL-c (mg/dL)	114.1 ± 46.9	84.0 ± 31.8	85.9 ± 38.1	125.7 ± 63.5	83.8 ± 28.5	85.0 ± 27.4
HDL-c (mg/dL)	46.4 ± 8.9	46.3 ± 11.8	49.3 ± 11.2	54.6 ± 14.0	54.2 ± 12.3	58.8 ± 11.8
TGL (mg/dL)	122.4 ± 57.1	125.0 ± 49.1	121.7 ± 71.0	127.0 ± 54.3	127.0 ± 61.5	118.4 ± 53.3
HbA1c (%)	6.2 ± 0.7	6.0 ± 0.8	5.6 ± 0.4	6.0 ± 0.8	5.8 ± 0.8	5.6 ± 0.5
Creatinine (mg/dL)	1.3 ± 0.2	1.3 ± 0.3	1.3 ± 0.2	1.1 ± 0.2	1.0 ± 0.3	1.1 ± 0.2

Urea (mg/dL)	38.6 ± 16.6	34.4 ± 12.5	39.8 ± 11.6	39.2 ± 13.0	31.8 ± 11.4	35.7 ± 10.9
ALT (U/L)	28.9 ± 10.0	30.2 ± 13.9	30.0 ± 13.5	26.0 ± 10.5	27.5 ± 14.8	22.0 ± 6.9
AST (U/L)	28.2 ± 11.8	33.3 ± 12.8	26.9 ± 11.1	28.6 ± 12.4	26.0 ± 8.3	25.3 ± 8.4
ALP (U/L)	52.0 ± 19.5	56.7 ± 18.6	53.2 ± 12.1	59.4 ± 22.0	61.0 ± 21.2	65.3 ± 17.0
GGT (U/L)	48.8 ± 19.3	44.0 ± 18.8	39.3 ± 16.2	42.2 ± 17.6	38.1 ± 18.9	29.2 ± 10.4
Albumin (g/dL)	4.1 ± 0.6	4.2 ± 0.3	4.1 ± 0.6	4.0 ± 0.6	4.0 ± 0.4	4.1 ± 0.7
Amylase (U/L)	73.8 ± 37.5	73.0 ± 27.6	72.7 ± 31.6	74.1 ± 24.4	65.2 ± 26.9	57.9 ± 27.1
Lipase (U/L)	49.5 ± 16.8	47.7 ± 11.8	38.8 ± 13.1	49.1 ± 17.0	45.8 ± 13.6	42.5 ± 14.0
Calcium (mg/dL)	6.8 ± 3.6	6.6 ± 2.9	6.9 ± 3.0	6.0 ± 2.6	6.6 ± 2.8	7.2 ± 3.7
Magnesium (mg/dL)	2.2 ± 0.8	2.1 ± 0.7	1.6 ± 0.7	2.2 ± 0.8	2.2 ± 0.9	1.5 ± 0.9
Phosphorus (mg/dL)	2.7 ± 1.2	2.9 ± 0.8	3.4 ± 1.0	2.9 ± 1.1	3.1 ± 1.0	3.4 ± 0.6
CRP (mg/dL)	7.4 ± 6.5	8.6 ± 6.1	5.9 ± 5.6	9.9 ± 9.1	7.1 ± 5.6	5.7 ± 4.4

Note: Data was described by mean and standard deviation (±). TC = total cholesterol; LDL-c = LDL cholesterol; HDL-c = HDL cholesterol; TGL = triglycerides; HbA1c = glycated hemoglobin; ALT = alanine aminotransferase; AST = aspartate aminotransferase; ALP = alkaline phosphatase; GGT = gamma-glutamyl transferase; CRP = C-reactive protein.

Supplementary Table S4 presents anthropometry and body composition responses pre-test, after 8, and after 16 weeks of intervention in the three COVID-19 male survivors' groups considering the symptoms (mild, moderate, and severe/critical).

Supplementary Table S4. Anthropometry and body composition responses pre-test, after 8, and after 16 weeks of intervention in the three COVID-19 male survivors' groups considering the symptoms (mild, moderate, and severe/critical).

Variables	**Mild (*n* = 18)**			**Moderate (*n* = 4)**			**Severe/critical (*n* = 10)**		
	**Pre-test**	**Post-8W**	**Post-16W**	**Pre-test**	**Post-8W**	**Post-16W**	**Pre-test**	**Post-8W**	**Post-16W**
Body mass (kg)	92.8 ± 20.5	92.4 ± 20.2	89.1 ± 26.3	91.5 ± 14.2	90.5 ± 13.6	90.1 ± 14.3	107.6 ± 23.2	106.9 ± 22.8	106.6 ± 22.0
BMI (kg/m^2)	30.4 ± 5.7	30.3 ± 5.6	30.2 ± 5.5	30.6 ± 4.7	30.3 ± 4.7	30.1 ± 4.8	33.3 ± 5.4	33.0 ± 5.1	32.7 ± 4.8
AC (cm)	105.1 ± 15.1	104.3 ± 15.2	103.7 ± 15.3	107.1 ± 11.3	105.3 ± 11.1	104.5 ± 11.7	114.9 ± 15.4	112.1 ± 12.5	114.2 ± 14.0
FMM (kg)	60.5 ± 9.7	60.7 ± 10.2	61.8 ± 10.8	58.2 ± 10.0	56.9 ± 10.0	56.9 ± 10.6	64.1 ± 10.5	61.1 ± 13.8	65.7 ± 12.0
SMM (kg)	33.8 ± 5.8	34.0 ± 6.0	34.7 ± 6.4	32.7 ± 6.1	31.8 ± 6.2	31.9 ± 6.6	35.9 ± 6.3	36.0 ± 6.4	36.9 ± 7.2
FM (kg)	32.3 ± 13.8	31.7 ± 12.8	32.8 ± 14.1	33.3 ± 8.4	33.6 ± 9.2	33.2 ± 7.2	43.6 ± 14.7	42.8 ± 14.3	40.9 ± 11.9
BFP (%)	33.6 ± 7.8	33.2 ± 7.4	32.1 ± 8.2	36.3 ± 6.1	37.0 ± 7.2	36.9 ± 5.6	41.5 ± 4.1	39.1 ± 7.2	37.8 ± 6.1

Note: Data was described by the mean and standard deviation (±). W = weeks; BMI = body mass index; AC = abdominal circumference; FFM = fat-free mass; SMM = musculoskeletal mass; FM = fat mass; BFP = body fat percentage.

Supplementary Table S5 presents anthropometry and body composition responses pre-test, after 8, and after 16 weeks of intervention in the three COVID-19 female survivors' groups considering the symptoms (mild, moderate, and severe/critical).

Supplementary Table S5. Anthropometry and body composition responses pre-test, after 8, and after 16 weeks of intervention in the three COVID-19 female survivors' groups considering the symptoms (mild, moderate, and severe/critical).

Variables	**Mild (*n* = 13)**			**Moderate (*n* = 9)**			**Severe/critical (*n* = 5)**		
	**Pre-test**	**Post-8W**	**Post-16W**	**Pre-test**	**Post-8W**	**Post-16W**	**Pre-test**	**Post-8W**	**Post-16W**
Body mass (kg)	73.0 ± 10.0	73.6 ± 10.0	73.6 ± 9.9	75.6 ± 15.1	76.2 ± 15.3	76.5 ± 15.4	85.2 ± 15.3	72.0 ± 22.6	84.1 ± 26.3

BMI (kg/m²)	28.4 ± 3.2	28.6 ± 3.2	28.6 ± 3.3	31.2 ± 6.7	31.4 ± 6.7	31.5 ± 7.0	33.0 ± 5.7	32.6 ± 5.4	32.6 ± 5.5
AC (cm)	96.1 ± 9.2	95.3 ± 8.1	96.0 ± 8.3	99.4 ± 15.4	98.7 ± 14.9	98.6 ± 15.4	103.1 ± 11.4	101.8 ± 8.4	99.2 ± 7.6
FMM (kg)	42.6 ± 5.7	43.2 ± 5.2	42.8 ± 5.5	40.9 ± 4.6	41.0 ± 4.7	41.2 ± 4.8	44.5 ± 5.4	44.8 ± 4.6	46.0 ± 3.9
SMM (kg)	23.2 ± 3.4	23.5 ± 3.1	23.3 ± 3.3	22.1 ± 2.7	22.2 ± 2.8	22.4 ± 2.8	24.5 ± 3.1	24.7 ± 2.7	25.4 ± 2.3
FM (kg)	30.4 ± 7.5	30.5 ± 7.7	30.8 ± 7.1	34.7 ± 12.0	35.3 ± 12.2	35.2 ± 12.2	40.6 ± 11.3	39.2 ± 10.8	328.1 ± 10.9
BFP (%)	41.3 ± 6.2	41.0 ± 5.9	41.6 ± 5.4	44.6 ± 8.3	44.9 ± 8.5	44.7 ± 8.5	47.1 ± 5.5	46.1 ± 5.3	44.7 ± 5.4

Note: Data was described by the mean and standard deviation (±). W = weeks; BMI = body mass index; AC = abdominal circumference; FFM = fat-free mass; SMM = musculoskeletal mass; FM = fat mass; BFP = body fat percentage.

Supplementary Table S6 presents the physical and cardiorespiratory fitness responses pre-test, after 8 and 16 weeks of intervention in the three COVID-19 male survivors' groups considering the symptoms (mild, moderate, and severe/critical).

Supplementary Table S6. Physical and cardiorespiratory fitness responses pre-test, after 8 and 16 weeks of intervention in the three COVID-19 male survivors' groups considering the symptoms (mild, moderate, and severe/critical).

Variables	**Mild (*n* = 18)**			**Moderate (*n* = 4)**			**Severe/critical (*n* = 10)**		
	**Pre-test**	**Post-8W**	**Post-16W**	**Pre-test**	**Post-8W**	**Post-16W**	**Pre-test**	**Post-8W**	**Post-16W**
MIHS-R (kgf)	37.8 ± 8.4	42.8 ± 8.7	41.0 ± 10.3	41.2 ± 14.6	39.8 ± 17.9	43.3 ± 14.1	39.5 ± 11.2	41.2 ± 10.1	42.0 ± 12.0
MIHS-L (kgf)	35.2 ± 8.4	40.3 ± 8.0	40.8 ± 10.1	32.8 ± 6.7	38.2 ± 17.9	42.6 ± 13.8	38.8 ± 12.0	38.6 ± 11.7	40.0 ± 13.3
Sit and reach (cm)	20.3 ± 9.5	22.2 ± 10.4	24.7 ± 9.2	24.5 ± 7.9	32.3 ± 4.0	31.2 ± 3.1	19.3 ± 9.7	20.4 ± 9.8	21.8 ± 8.9
MILTS (kg)	112.8 ± 29.3	126.2 ± 30.0	126.5 ± 28.8	91.7 ± 7.6	110.3 ± 23.2	101.5 ± 31.3	126.9 ± 51.4	123.0 ± 44.4	123.7 ± 47.2
Push-up (reps/min)	18.2 ± 10.3	23.4 ± 13.0	28.7 ± 15.2	8.8 ± 5.4	17.0 ± 5.5	23.0 ± 13.5	15.0 ± 7.8	15.0 ± 8.2	18.5 ± 9.5

Abdominal strength-endurance (reps/min)	19.7 ± 7.9	23.8 ± 9.2	30.9 ± 13.3	20.5 ± 8.4	25.8 ± 8.3	28.3 ± 3.3	16.3 ± 9.5	20.7 ± 9.9	20.0 ± 11.4
Sit and stand (reps/min)	17.3 ± 4.8	20.3 ± 7.0	21.7 ± 7.2	14.0 ± 3.2	22.3 ± 9.6	23.5 ± 4.7	16.0 ± 6.2	17.6 ± 6.9	17.4 ± 5.3
6MWT									
VO_2peak (mL/kg/min)	17.8 ± 3.8	19.1 ± 4.1	19.9 ± 4.2	18.8 ± 6.9	19.9 ± 6.4	19.8 ± 6.0	17.1 ± 4.4	17.5 ± 4.5	17.6 ± 3.7
Distance (m)	547.5 ± 74.9	622.8 ± 89.7	627.2 ± 108.0	577.6 ± 91.1	615.1 ± 83.9	610.9 ± 50.4	559.7 ± 109.2	584.9 ± 118.3	567.6 ± 94.2
SpO_2 final (%)	95.6 ± 2.1	96.1 ± 1.4	95.3 ± 2.6	96.5 ± 1.3	97.3 ± 1.5	95.3 ± 1.3	93.1 ± 3.1	94.1 ± 2.3	95.0 ± 2.9
Final heart rate (bpm)	128.7 ± 24.7	132.7 ± 20.8	132.9 ± 24.7	133.3 ± 34.2	142.5 ± 37.0	138.3 ± 33.2	131.8 ± 28.9	125.7 ± 34.5	135.1 ± 22.5
SBP pre-test (mmHg)	126.7 ± 12.4	124.7 ± 8.2	121.9 ± 12.2	132.5 ± 15.0	136.5 ± 7.9	142.5 ± 17.1	126.7 ± 13.2	134.0 ± 16.5	132.0 ± 16.2
SBP final (mmHg)	146.7 ± 17.2	150.0 ± 9.7	136.0 ± 16.1	137.5 ± 27.5	147.5 ± 22.2	148.5 ± 19.6	142.2 ± 21.1	147.0 ± 10.6	149.9 ± 10.4
DBP pre-test (mmHg)	80.0 ± 10.9	82.5 ± 10.7	73.1 ± 9.5	87.5 ± 9.6	81.8 ± 3.5	78.3 ± 16.1	81.1 ± 12.7	88.0 ± 13.2	80.0 ± 9.4
DBP final (mmHg)	84.4 ± 10.4	86.3 ± 12.0	78.1 ± 11.7	87.5 ± 20.6	90.0 ± 8.2	82.5 ± 5.0	85.6 ± 15.9	88.0 ± 12.3	82.8 ± 12.9

Note: Data was described by mean and standard deviation (±). W = weeks; SBP = systolic blood pressure; DBP = diastolic blood pressure; MIHS = maximal isometric handgrip strength; R = right side; L = left side; MILTS = maximal isometric lumbar-traction strength; 6MWT = 6-minute walk test; VO_2 peak = peak oxygen consumption.

Supplementary Table S7 presents the physical and cardiorespiratory fitness responses pre-test, after 8 and 16 weeks of intervention in the three COVID-19 female survivors' groups considering the symptoms (mild, moderate, and severe/critical).

Supplementary Table S7. Physical and cardiorespiratory fitness responses pre-test, after 8 and 16 weeks of intervention in the three COVID-19 female survivors' groups considering the symptoms (mild, moderate, and severe/critical).

Variables	**Mild (*n* = 13)**			**Moderate (*n* = 9)**			**Severe/critical (*n* = 5)**		
	**Pre-test**	**Post-8W**	**Post-16W**	**Pre-test**	**Post-8W**	**Post-16W**	**Pre-test**	**Post-8W**	**Post-16W**
MIHS-R (kgf)	37.8 ± 8.4	42.8 ± 8.7	41.0 ± 10.3	41.2 ± 14.6	39.8 ± 17.9	43.3 ± 14.1	39.5 ± 11.2	41.2 ± 10.1	42.0 ± 12.0
MIHS-L (kgf)	35.2 ± 8.4	40.3 ± 8.0	40.8 ± 10.1	32.8 ± 6.7	38.2 ± 17.9	42.6 ± 13.8	38.8 ± 12.0	38.6 ± 11.7	40.0 ± 13.3
Sit and reach (cm)	20.3 ± 9.5	22.2 ± 10.4	24.7 ± 9.2	24.5 ± 7.9	32.3 ± 4.0	31.2 ± 3.1	19.3 ± 9.7	20.4 ± 9.8	21.8 ± 8.9
MILTS (kg)	112.8 ± 29.3	126.2 ± 30.0	126.5 ± 28.8	91.7 ± 7.6	110.3 ± 23.2	101.5 ± 31.3	126.9 ± 51.4	123.0 ± 44.4	123.7 ± 47.2
Push-up (reps/min)	18.2 ± 10.3	23.4 ± 13.0	28.7 ± 15.2	8.8 ± 5.4	17.0 ± 5.5	23.0 ± 13.5	15.0 ± 7.8	15.0 ± 8.2	18.5 ± 9.5
Abdominal strength-endurance (reps/min)	19.7 ± 7.9	23.8 ± 9.2	30.9 ± 13.3	20.5 ± 8.4	25.8 ± 8.3	28.3 ± 3.3	16.3 ± 9.5	20.7 ± 9.9	20.0 ± 11.4
Sit and stand (reps/min)	17.3 ± 4.8	20.3 ± 7.0	21.7 ± 7.2	14.0 ± 3.2	22.3 ± 9.6	23.5 ± 4.7	16.0 ± 6.2	17.6 ± 6.9	17.4 ± 5.3
6MWT									
VO_2peak (mL/kg/min)	17.8 ± 3.8	19.1 ± 4.1	19.9 ± 4.2	18.8 ± 6.9	19.9 ± 6.4	19.8 ± 6.0	17.1 ± 4.4	17.5 ± 4.5	17.6 ± 3.7
Distance (m)	547.5 ± 74.9	622.8 ± 89.7	627.2 ± 108.0	577.6 ± 91.1	615.1 ± 83.9	610.9 ± 50.4	559.7 ± 109.2	584.9 ± 118.3	567.6 ± 94.2
SpO_2 final (%)	95.6 ± 2.1	96.1 ± 1.4	95.3 ± 2.6	96.5 ± 1.3	97.3 ± 1.5	95.3 ± 1.3	93.1 ± 3.1	94.1 ± 2.3	95.0 ± 2.9
Final heart rate (bpm)	128.7 ± 24.7	132.7 ± 20.8	132.9 ± 24.7	133.3 ± 34.2	142.5 ± 37.0	138.3 ± 33.2	131.8 ± 28.9	125.7 ± 34.5	135.1 ± 22.5
SBP pre-test (mmHg)	126.7 ± 12.4	124.7 ± 8.2	121.9 ± 12.2	132.5 ± 15.0	136.5 ± 7.9	142.5 ± 17.1	126.7 ± 13.2	134.0 ± 16.5	132.0 ± 16.2

SBP final (mmHg)	146.7 ± 17.2	150.0 ± 9.7	136.0 ± 16.1	137.5 ± 27.5	147.5 ± 22.2	148.5 ± 19.6	142.2 ± 21.1	147.0 ± 10.6	149.9 ± 10.4
DBP pre-test (mmHg)	80.0 ± 10.9	82.5 ± 10.7	73.1 ± 9.5	87.5 ± 9.6	81.8 ± 3.5	78.3 ± 16.1	81.1 ± 12.7	88.0 ± 13.2	80.0 ± 9.4
DBP final (mmHg)	84.4 ± 10.4	86.3 ± 12.0	78.1 ± 11.7	87.5 ± 20.6	90.0 ± 8.2	82.5 ± 5.0	85.6 ± 15.9	88.0 ± 12.3	82.8 ± 12.9

Note: Data was described by mean and standard deviation (±). W = weeks; SBP = systolic blood pressure; DBP = diastolic blood pressure; MIHS = maximal isometric handgrip strength; R = right side; L = left side; MILTS = maximal isometric lumbar-traction strength; 6MWT = 6-minute walk test; VO_2 peak = peak oxygen consumption.

Supplementary Table S8 presents the Biochemical parameters responses pre-test, after 8, and after 16 weeks of intervention in the three male COVID-19 survivors' groups.

Supplementary Table S8. Biochemical parameters responses pre-test, after 8, and after 16 weeks of intervention in the three male COVID-19 survivors' groups.

Variables	**Mild (*n* = 18)**			**Moderate (*n* = 4)**			**Severe/critical (*n* = 10)**		
	**Pre-test**	**Post-8W**	**Post-16W**	**Pre-test**	**Post-8W**	**Post-16W**	**Pre-test**	**Post-8W**	**Post-16W**
TC (mg/dL)	176.3 ± 38.7	156.2 ± 36.2	164.7 ± 35.6	185.3 ± 44.3	133.5 ± 37.5	146.3 ± 34.4	191.2 ± 49.8	167.6 ± 29.0	161.0 ± 47.9
LDL-c (mg/dL)	109.7 ± 38.1	82.5 ± 32.9	87.7 ± 30.1	126.2 ± 47.9	61.7 ± 26.9	72.4 ± 32.7	117.2 ± 63.0	95.6 ± 28.8	88.0 ± 53.3
HDL-c (mg/dL)	46.9 ± 10.6	49.5 ± 10.6	52.5 ± 9.7	42.7 ± 5.9	40.0 ± 17.4	39.5 ± 11.2	47.1 ± 5.8	42.9 ± 10.8	47.6 ± 12.2
TGL (mg/dL)	117.5 ± 53.7	121.3 ± 52.6	107.5 ± 47.8	112.0 ± 44.1	111.0 ± 34.6	113.7 ± 74.1	135.4 ± 69.6	136.9 ± 47.8	148.3 ± 98.8
HbA1c (%)	6.2 ± 0.6	6.0 ± 0.8	5.6 ± 0.4	6.5 ± 1.4	6.3 ± 1.0	5.6 ± 0.2	6.0 ± 0.3	5.8 ± 0.5	5.7 ± 0.4
Creatinine (mg/dL)	1.3 ± 0.2	1.3 ± 0.3	1.3 ± 0.2	1.3 ± 0.3	1.3 ± 0.2	1.2 ± 0.2	1.3 ± 0.2	1.4 ± 0.2	1.4 ± 0.1
Urea (mg/dL)	35.1 ± 14.6	35.7 ± 13.9	43.8 ± 12.4	51.8 ± 28.4	30.5 ± 15.7	35.5 ± 6.2	39.5 ± 13.2	33.6 ± 8.1	34.4 ± 9.4
ALT (U/L)	27.3 ± 10.0	28.9 ± 14.5	34.9 ± 14.0	34.5 ± 14.6	34.3 ± 18.6	26.5 ± 9.5	29.5 ± 8.0	30.9 ± 12.0	22.4 ± 10.2
AST (U/L)	28.1 ± 8.8	33.3 ± 11.0	29.1 ± 9.9	22.8 ± 4.7	42.5 ± 25.8	22.5 ± 11.2	30.5 ± 17.1	29.5 ± 7.8	24.8 ± 13.3
ALP (U/L)	48.8 ± 17.6	57.1 ± 19.9	55.5 ± 11.7	51.0 ± 33.1	52.5 ± 28.2	48.8 ± 20.4	58.1 ± 17.2	57.7 ± 12.7	51.3 ± 9.2

GGT (U/L)	48.6 ± 20.6	43.9 ± 20.5	40.2 ± 17.3	40.8 ± 13.7	46.8 ± 23.7	44.8 ± 21.4	52.5 ± 19.4	43.2 ± 15.3	35.5 ± 12.5
Albumin (g/dL)	4.2 ± 0.7	4.3 ± 0.3	4.1 ± 0.6	4.1 ± 0.8	4.1 ± 0.2	3.9 ± 0.6	4.0 ± 0.5	4.0 ± 0.3	4.1 ± 0.7
Amylase (U/L)	83.3 ± 41.4	81.3 ± 23.0	71.9 ± 28.3	50.3 ± 31.1	57.3 ± 55.7	61.8 ± 19.0	66.0 ± 28.2	64.4 ± 16.1	78.4 ± 41.5
Lipase (U/L)	52.0 ± 19.2	46.7 ± 14.6	36.6 ± 12.4	50.5 ± 17.6	46.8 ± 8.7	44.8 ± 9.2	44.6 ± 11.6	49.9 ± 6.7	40.2 ± 15.6
Calcium (mg/dL)	7.0 ± 3.9	6.0 ± 2.4	6.8 ± 2.6	6.8 ± 3.1	7.5 ± 3.6	9.2 ± 4.6	6.6 ± 3.6	7.5 ± 3.6	6.1 ± 2.6
Magnesium (mg/dL)§	2.1 ± 0.5	2.0 ± 0.7	1.4 ± 0.7	2.2 ± 1.7	2.1 ± 0.8	2.1 ± 0.8	2.4 ± 0.7	1.8 ± 0.6	1.4 ± 0.7
Phosphorus (mg/dL)	2.6 ± 0.8	3.0 ± 0.8	3.6 ± 1.2	1.9 ± 0.4	2.7 ± 0.5	3.3 ± 0.2	3.2 ± 1.7	2.8 ± 0.7	3.0 ± 0.7
CRP (mg/dL)	6.5 ± 6.6	6.6 ± 4.7	5.5 ± 5.5	3.9 ± 4.0	16.1 ± 9.8	8.7 ± 6.0	10.4 ± 6.6	10.1 ± 5.2	5.4 ± 6.0

Note: Data was described by mean and standard deviation (±). TC = total cholesterol; LDL-c = LDL cholesterol; HDL-c = HDL cholesterol; TGL = triglycerides; HbA1c = glycated hemoglobin; ALT = alanine aminotransferase; AST = aspartate aminotransferase; ALP = alkaline phosphatase; GGT = gamma-glutamyl transferase; CRP = C-reactive protein.

Supplementary Table S9 presents the Biochemical parameters responses pre-test, after 8, and after 16 weeks of intervention in the three female COVID-19 survivors' groups.

Supplementary Table S9. Biochemical parameters responses pre-test, after 8, and after 16 weeks of intervention in the three female COVID-19 survivors' groups.

Variables	**Mild (*n* = 13)**			**Moderate (*n* = 9)**			**Severe/critical (*n* = 5)**		
	**Pre-test**	**Post-8W**	**Post-16W**	**Pre-test**	**Post-8W**	**Post-16W**	**Pre-test**	**Post-8W**	**Post-16W**
TC (mg/dL)	190.3 ± 59.3	176.2 ± 30.0	173.9 ± 34.6	203.2 ± 62.1	159.8 ± 44.5	165.3 ± 31.9	183.8 ± 86.4	140.4 ± 43.2	148.4 ± 29.6
LDL-c (mg/dL)	132.8 ± 66.8	94.8 ± 19.6	88.5 ± 29.8	123.9 ± 58.6	79.7 ± 36.3	86.5 ± 25.7	110.4 ± 74.2	62.7 ± 22.1	73.0 ± 25.9
HDL-c (mg/dL)	53.3 ± 15.2	56.4 ± 13.4	60.8 ± 11.8	59.3 ± 14.1	54.5 ± 12.5	60.3 ± 12.7	49.7 ± 9.9	48.1 ± 8.1	50.7 ± 8.4

TGL (mg/dL)	118.2 ± 46.8	127.9 ± 58.0	123.9 ± 62.2	137.8 ± 64.8	112.3 ± 50.8	103.8 ± 37.4	130.6 ± 61.0	151.0 ± 90.2	130.2 ± 57.2
HbA1c (%)	6.0 ± 0.6	5.8 ± 0.6	5.5 ± 0.4	6.2 ± 1.1	5.9 ± 1.1	5.7 ± 0.5	5.5 ± 0.7	5.4 ± 0.9	5.5 ± 0.6
Creatinine (mg/dL)	1.2 ± 0.2	1.1 ± 0.2	1.0 ± 0.2	1.1 ± 0.2	0.9 ± 0.4	1.1 ± 0.2	1.1 ± 0.2	1.1 ± 0.1	1.3 ± 0.2
Urea (mg/dL)	42.9 ± 16.8	34.4 ± 11.7	37.5 ± 8.9	36.0 ± 7.7	29.4 ± 13.3	37.7 ± 13.5	35.2 ± 6.9	29.6 ± 6.3	25.0 ± 3.9
ALT (U/L)	27.7 ± 10.8	26.0 ± 11.5	20.8 ± 6.7	21.8 ± 9.3	22.7 ± 9.2	23.2 ± 8.6	29.2 ± 11.3	40.0 ± 24.4	22.8 ± 4.1
AST (U/L)	31.3 ± 12.6	23.5 ± 6.7	24.4 8.1	22.4 ± 10.7	30.3 ± 10.5	27.2 ± 9.9	32.4 ± 12.9	33.3 ± 5.6	24.2 ± 7.2
ALP (U/L)	54.8 ± 22.2	63.8 ± 23.4	58.8 ± 12.2	63.7 ± 17.8	55.4 ± 18.0	77.0 ± 18.5	64.0 ± 29.8	63.6 ± 23.0	61.0 ± 16.9
GGT (U/L)	41.5 ± 22.3	33.4 ± 16.6	28.9 ± 12.0	45.1 ± 14.4	41.3 ± 19.1	32.9 ± 10.0	39.0 ± 7.4	44.4 ± 25.1	24.0 ± 3.2
Albumin (g/dL)	4.1 ± 0.6	3.8 ± 0.4	3.9 ± 0.5	3.8 ± 0.7	4.1 ± 0.5	4.1 ± 0.7	4.1 ± 0.2	4.1 ± 0.2	4.3 ± 0.9
Amylase (U/L)	82.2 ± 22.4	70.4 ± 24.9	56.2 ± 29.3	72.3 ± 27.1	59.9 ± 21.1	53.1 ± 17.7	56.2 ± 16.5	61.2 ± 42.3	70.8 ± 36.1
Lipase (U/L)	54.3 ± 17.4	49.1 ± 7.5	40.9 ± 10.8	42.8 ± 17.0	37.4 ± 15.7	37.4 ± 15.9	46.5 ± 13.3	52.8 ± 16.0	55.6 ± 11.1
Calcium (mg/dL)	5.6 ± 3.1	6.5 ± 2.6	6.9 ± 3.7	5.7 ± 2.3	6.9 ± 3.0	8.6 ± 3.3	7.4 ± 3.9	6.3 ± 3.4	5.2 ± 3.9
Magnesium (mg/dL)§	2.3 ± 0.8	2.0 ± 0.9	1.4 ± 0.6	2.1 ± 1.0	1.8 ± 0.7	1.9 ± 1.2	2.3 ± 0.5	3.1 ± 0.7	1.4 ± 0.3
Phosphorus (mg/dL)	2.4 ± 0.9	3.1 ± 1.2	3.5 ± 0.8	3.4 ± 1.0	3.0 ± 0.6	3.6 ± 0.2	3.1 ± 1.3	3.3 ± 0.9	2.7 ± 0.3
CRP (mg/dL)	5.5 ± 7.6	5.5 ± 5.5	4.6 ± 3.1	11.5 ± 9.0	5.8 ± 2.8	4.6 ± 3.8	18.5 ± 6.5	12.2 ± 6.4	10.3 ± 5.7

Note: Data was described by mean and standard deviation (±). TC = total cholesterol; LDL-c = LDL cholesterol; HDL-c = HDL cholesterol; TGL = triglycerides; HbA1c = glycated hemoglobin; ALT = alanine aminotransferase; AST = aspartate aminotransferase; ALP = alkaline phosphatase; GGT = gamma-glutamyl transferase; CRP = C-reactive protein.
